# Screening for Diguanylate Cyclase (DGC) Inhibitors Mitigating Bacterial Biofilm Formation

**DOI:** 10.3389/fchem.2020.00264

**Published:** 2020-04-21

**Authors:** Kyu Hong Cho, R. Grant Tryon, Jeong-Ho Kim

**Affiliations:** ^1^Department of Biology, Indiana State University, Terre Haute, IN, United States; ^2^Department of Biology and Chemistry, Liberty University, Lynchburg, VA, United States

**Keywords:** biofilm, diguanylate cyclases, DGC inhibitors, high throughput screening, DGC activity assay

## Abstract

The majority of bacteria in the natural environment organize themselves into communal biofilms. Biofilm formation benefits bacteria conferring resistance to harmful molecules (e.g., antibiotics, disinfectants, and host immune factors) and coordinating their gene expression through quorum sensing (QS). A primary signaling molecule promoting bacterial biofilm formation is the universal second messenger cyclic di-GMP. This dinucleotide predominantly controls the gene expression of motility, adhesins, and capsule production to coordinate biofilm formation. Cyclic di-GMP is synthesized by diguanylate cyclases (DGCs) that have a GGDEF domain and is degraded by phosphodiesterases (PDEs) containing either an EAL or an HD-GYP domain. Since high cellular c-di-GMP concentrations are correlated with promoting the ability of bacteria to form biofilms, numerous research endeavors to identify chemicals capable of inhibiting the c-di-GMP synthesis activity of DGCs have been performed in order to inhibit bacterial biofilm formation. This review describes currently identified chemical inhibitors that disturb the activity of DGCs and the methods of screening and assay for their discovery.

## Introduction

### Bacterial Biofilms and Human Health

Biofilms are sessile multicellular bacterial communities encased in a three-dimensional meshwork formed by extracellular polymeric substances (EPSs) (Roy et al., 2018). EPSs consisting of polysaccharides, proteinaceous filaments, and/or nucleic acids can compose as much as 90% of biofilm's biomass (Limoli et al., 2015). EPSs can provide protection against mechanical stress by the formation of a protective physical barrier and control the diffusion of signaling molecules, nutrients, and toxic compounds (Morgan et al., 2014). Extensive intercellular communication and interactions have been observed within biofilms through quorum sensing (QS), which uses diffusible molecules known as autoinducers to regulate population behaviors (Li and Tian, 2012). QS appears to participate in the development of biofilm formation (Lin Chua et al., 2017; Kim et al., 2018). For example, N-acyl homoserine lactone (AHL) QS systems observed in *P. aeruginosa* biofilms were identified as mechanisms by which extracellular DNA (eDNA) was released while *P. aeruginosa* produced EPSs.

Chronic infections such as contamination of artificial medical implants, otitis media, chronic healing wounds, and lung pneumonia of cystic fibrosis patients are mostly associated with bacterial biofilm formation (Bjarnsholt, 2013). It is estimated that nineteen million annual infections are due to biofilm-based infections in the United States (Wolcott et al., 2010). Biofilm formation promotes increased antibiotic tolerance up to ~1,000 times greater than that observed in planktonic bacteria (Ito et al., 2009). Besides, biofilms resist host immune defense strategies, such as mechanical clearance, complement-mediated killing, antibody recognition, and phagocytosis (Domenech et al., 2013). Often, biofilm-based infections cannot be comprehensively treated due to ineffective antibiotic therapy (Sambanthamoorthy et al., 2012).

### c-di-GMP Signaling Systems Control Bacterial Biofilm Formation

c-di-GMP (bis-(3′-5′)-cyclic dimeric guanosine monophosphate or cyclic diguanylate monophosphate) is one of dinucleotide second messengers in bacteria. It was discovered in 1987 while researchers were studying cellulose synthesis in *Gluconoacetobacter xylinus* (Ross et al., 1987). Further studies revealed that it controls various cellular functions including EPS synthesis and secretion, flagellar motility, adhesion, cell cycle initiation and regulation, and virulence factor synthesis in bacteria (Caly et al., 2015; Kim et al., 2018). The main common role of c-di-GMP signaling in diverse bacteria is to regulate bacterial lifestyle by controlling the transition of bacteria between a planktonic lifestyle and a biofilm lifestyle (Chua et al., 2014). Generally, high c-di-GMP content in bacterial cells reduces their motility by inhibiting flagella assembly and increases the synthesis of the EPS matrix, resulting in biofilm formation. Low intracellular c-di-GMP concentration increases bacterial motility and disperse biofilms (Hengge, 2009; Lee et al., 2010; Chua et al., 2015; Gao et al., 2017). Since c-di-GMP signaling systems are highly conserved in bacteria but not in eukaryotic organisms, and c-di-GMP promotes biofilm formation, enzymes associated with its metabolism are attractive targets for the interference with bacterial biofilm formation. Many important human pathogens whose biofilm formation ability plays a pivotal role in their virulence possess numerous c-di-GMP metabolizing enzymes, including *P. aeruginosa, Clostridium difficile, Salmonella typhimurium*, and *Vibrio cholerae* (Navarro et al., 2009; Solano et al., 2009; Antoniani et al., 2010; Bordeleau et al., 2011; Ha and O'Toole, 2015; Conner et al., 2017).

### Enzymes Involved in c-di-GMP Synthesis and Degradation

**Figure 1:**
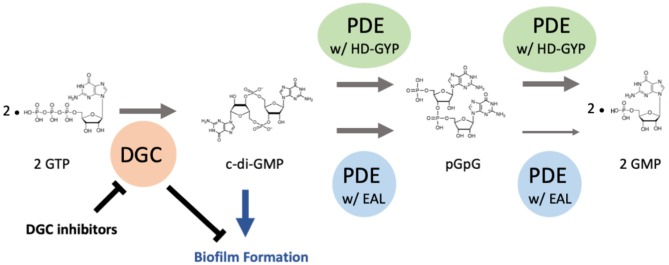
c-di-GMP metabolic pathway. Diguanylate cyclases (DGCs) with a GGDEF domain synthesize c-di-GMP, which is degraded by phosphodiesterases (PDEs) with an HD-GYP or an EAL domain. Phosphodiesterases degrade c-di-GMP to pGpG first then GMP. Phosphodiesterases with the EAL domain degrade pGpG to GMP slower than those with the HD-GYP domain. Generally, c-di-GMP promotes bacterial biofilm formation. When DGC enzyme activity is lowered by chemical inhibitors, less amount of c-di-GMP is produced, then biofilm formation by bacteria is discouraged.

The intracellular levels of c-di-GMP at a given time are determined by the combination of activities of diguanylate cyclases (DGCs) and phosphodiesterases (PDEs) (Figure 1) (Christen et al., 2006). Both classes of enzymes possess several N-terminal sensory domains allowing a prompt response to various environmental stimuli, including the presence of oxygen, light, nitric oxide, and other specific compounds (Gomelsky and Klug, 2002; Tuckerman et al., 2009; Plate and Marletta, 2012). DGCs and PDEs are often physically linked together even though they perform opposing reactions, but the catalytic function of one of them has usually lost and instead has gained a function to control the protein activity. Up to now, only a few proteins have been found to possess both c-di-GMP synthesizing and degradative activities (Wirebrand et al., 2018).

Two molecules of guanosine triphosphate (GTP) are synthesized into c-di-GMP via the activity of a GGDEF domain located in DGCs (Christen et al., 2006). The GGDEF signature domain forms part of the active site “A-site” where GTP is bound (one molecule of GTP substrate per monomer) (Chan et al., 2004). Structural analysis of a GGDEF domain protein PleD (a DGC of *Caulobacter crescentus*) showed that it binds dimeric c-di-GMP at an allosteric site “I-site” that is characterized by the RxxD motif (Chan et al., 2004). Subsequent structural determination of PleD and WspR (a DGC in *P. aeruginosa*) bound to c-di-GMP confirmed that the activity of the GGDEF domain is regulated by feedback inhibition, in which the binding of c-di-GMP to the I-site prevents the formation of an enzymatically active DGC dimer (Wassmann et al., 2007; De N et al., 2008). Thus, the binding of c-di-GMP at the I-site accounts for a strong non-competitive product inhibition, which establishes a limit on the cellular c-di-GMP concentration.

PDEs possessing either an EAL or an HD-GYP domain are responsible for degrading c-di-GMP (Caly et al., 2015). Generally, PDEs with the EAL domain degrade c-di-GMP into linear pGpG, and the ones with the HD-GYP domain degrade into two molecules of GMP (Povolotsky and Hengge, 2012; Sundriyal et al., 2014). The PDEs with the EAL domain predominantly produce pGpG from c-di-GMP first then GMP from pGpG very slowly, suggesting that the pGpG-degrading activity is irrelevant *in vivo* (Shanahan et al., 2013). The PDEs with the HD-GYP domain that contains a HHExxDGxxGYP motif are a subgroup of the HD superfamily of metal-dependent phosphohydrolases and convert c-di-GMP to GMP via the linear nucleotide pGpG (Dow et al., 2006; Stelitano et al., 2013a).

It is estimated that ~80% of human infections are caused by microbial biofilms (Hall-Stoodley et al., 2004), so there have been many elaborate scientific studies in order to inhibit the biofilm formation or destroy established biofilms. One of the promising ways is to develop inhibitory chemicals against di-guanylate cyclases (DGCs) that produce c-di-GMP that promotes biofilm formation in diverse bacteria. This paper reviews what DGC inhibitors have been found so far, what are their strength and weakness as biofilm inhibitors, and what screening methods have been used to find the inhibitors.

## DGC Inhibitors

Since high cellular c-di-GMP concentrations promote biofilm formation and maintenance, the main target enzyme in the c-di-GMP metabolism for the development of biofilm inhibitors has been DGCs. Thus, there have been many studies to find effective inhibitors against DGCs. In this section, DGC inhibitors that have been discovered so far, and their screening and assay methods are described, which is summarized in Table 1.

**Table 1:** Identified chemical inhibitors against Diguanylate Cyclases (DGCs).

**Name of chemical**	**Mode of action**	**Screening and DGC activity assay methods**	**Chemical library used for screening**	**References**
**Natural molecules**				
Glycosylated Triterpenoid Saponin	Non-competitive inhibition	*In vitro* enzyme activity assay analyzed by TLC (thin layer chromatograph) followed by autoradiography	Plant extract	Ohana et al., 1998
**c-di-GMP analogs**				
2′-F-c-di-GMP	Non-competitive inhibition binding to the DGC I-site	*In vitro* enzyme activity assay analyzed by TLC followed by autoradiography	Three c-di-GMP analogs chemically synthesized	Zhou et al., 2013
c-di-Inosinylic Acid	Non-competitive inhibition binding to the DGC I-site	*In vitro* enzyme activity assay analyzed by HPLC (high performance liquid chromatography)	Five c-di-GMP analogs chemically synthesized	Ching et al., 2010
Triazole-Linked Analog DCI058	Non-competitive inhibition binding to the DGC I-site	*In vitro* enzyme activity assay analyzed by circular dichroism (CD) spectroscopy	16 c-di-GMP analogs chemically synthesized	Fernicola et al., 2015
**GTP analogs**				
MANT-GTP and MANT-GTPγS	Unknown, probably competitive inhibition	*In vitro* enzyme activity assay analyzed by HPLC-MS/MS	8 NTP derivatives	Spangler et al., 2011
TNP-GTP	Unknown, probably competitive inhibition	*In vitro* enzyme activity assay analyzed by HPLC-MS/MS	8 NTP derivatives	Spangler et al., 2011
**Small synthetic molecules**				
Ebselen	Cysteine residue modification near the I-site	DRaCALA (Differential Radial Capillary Action of Ligand Assay)	NIH clinical collection 1 (NCC1) library	Lieberman et al., 2014
Catechol-containing Sulfonohydrazide compounds	Competitive inhibition of active site	*In silico* (3-D Pharmacophore) prediction followed by *in vitro* enzyme activity assay	The purchasable subset of the ZINC database (~2.3 × 10^7^ compounds)	Fernicola et al., 2016
Sulfasalazine	Competitive inhibition of active site	*In silico* prediction with OpenEye Scientific Software followed by *in vitro* enzyme activity assay	1,500 FDA-approved drugs in the DrugBank database	Wiggers et al., 2018
Eprosartan	Competitive inhibition of active site	*In silico* prediction with OpenEye Scientific Software followed by *in vitro* enzyme activity assay	1,500 FDA-approved drugs in the DrugBank database	Wiggers et al., 2018
N-(4-anilinophenyl)benzamide (aka. DI-3)	Unknown	Screening with an *in vivo* luciferase reporter assay followed by an *in vitro* enzyme activity assay	66,000 compounds/natural product extracts at the Center for Chemical Genomics at the University of Michigan	Sambanthamoorthy et al., 2012
[2- oxo-2-(2-oxopyrrolidin-1-yl)ethyl] 1,3-benzothiazole-6-carboxylate	Non-competitive inhibition binding to the DGC I-site	FRET (Foster resonance energy transfer)-based *in vitro* enzyme activity assay	27,502 commercially available small molecules	Christen et al., 2019
4-(2,5-dimethylphenoxy)-N-(4-morpholin-4-ylphenyl)butanamide	Non-competitive inhibition binding to the DGC I-site	FRET (Foster resonance energy transfer)-based *in vitro* enzyme activity assay	27,502 commercially available small molecules derived from chemical libraries at the Institute of Chemical Biology at Harvard University	Christen et al., 2019
N′-((1E)-{4-ethoxy-3-[(8-oxo-1,5,6,8-tetrahydro-2H-1,5-methanopyrido[1,2-a] [1,5]diazocin-3(4H)-yl)methyl]phenyl}methylene)-3,4,5-trihydroxybenzohydrazide (aka. LP-3134)	Competitive inhibition of active site	*In silico* (3-D Pharmacophore) prediction followed by *in vitro* enzyme activity assay	A database of commercially available compounds	Sambanthamoorthy et al., 2014

### Natural Molecules

#### Glycosylated Triterpenoid Saponin (GTS)

**Figure 2:**
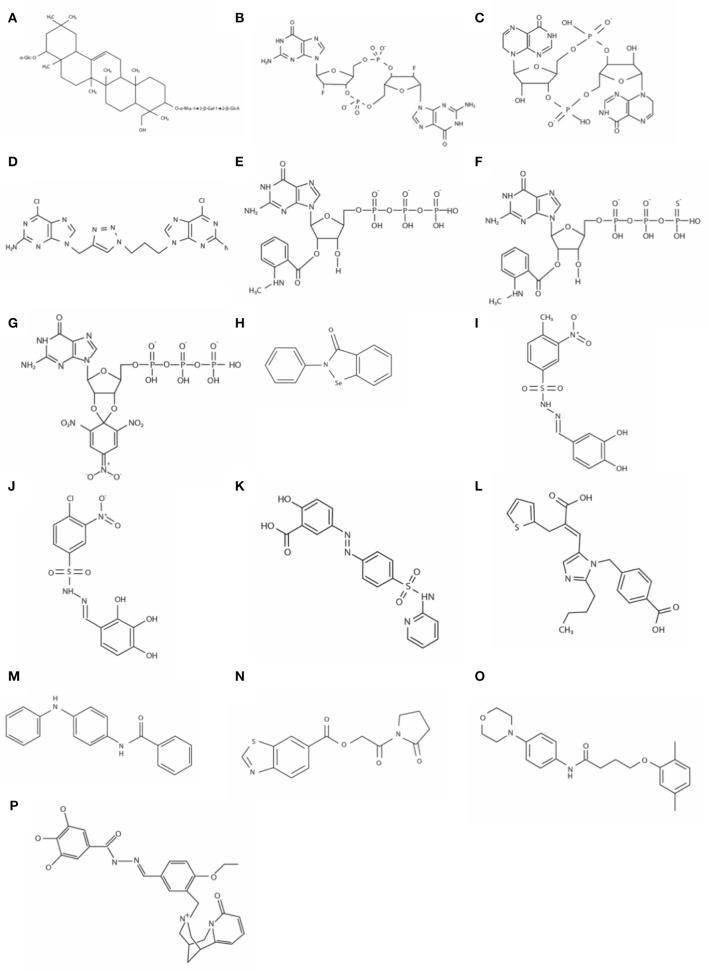
The structures of DGC inhibitors. **(A)** 3-o-α-L-rhamnopyronosyl-(12)-β-D-galactopyranosyl-(12)-β-D-gluconopyranosyl soyasapogenol B 22-o-α-D-glucopyranoside; **(B)** 2′-F-c-di-GMP; **(C)** c-di-inosinylic acid; **(D)** DC1058; **(E)** MANT-GTP; **(F)** MANT-GTPγS; **(G)** TNP-GTP; **(H)** Ebselen; **(I)** Amb2250087; **(J)** Amb379455; **(K)** Sulfasalazine; **(L)** Eprosartan; **(M)** N-(4-anilinophenyl)benzamide; **(N)** [2- oxo-2-(2-oxopyrrolidin-1-yl)ethyl] 1,3-benzothiazole-6-carboxylate; **(O)** 4-(2,5-dimethylphenoxy)-N-(4-morpholin-4-ylphenyl)butanamide; and **(P)** N′-((1E)-{4-ethoxy-3-[(8-oxo-1,5,6,8-tetrahydro-2H-1,5-methanopyrido[1,2-a] [1,5]diazocin-3(4H)-yl)methylphenyl}methylene)]-3,4,5-trihydroxybenzohydrazide, aka LP3134.

A unique triterpenoid saponin extracted from the pea plant (*Pisum sativum*) was identified as a potent inhibitor of a DGC in *Gluconoacetobacter xylinus* (formerly *Acetobacter xylinum*) (Ohana et al., 1998). Spectral analyses identified this compound as 3-o-α-L-rhamnopyronosyl-(12)-β-D-galactopyranosyl-(12)-β-D-gluconopyranosyl soyasapogenol B 22-o-α-D-glucopyranoside (Figure 2A). This family of compounds is widely distributed in higher plants, but its role in plants has not been elucidated yet. A compound with an identical or at least very similar structure is also produced by the cellulose synthesizing bacterium *G. xylinus*. This study suggests this type of saponin might be involved in cellulose synthesis in plants and the bacterium *G. xylinus*. The IC_50_ (50% inhibitory concentration) is about 5 μM *in vitro*. The GTS, however, does not inhibit DGC activity *in vivo*, probably due to non-permeable nature to the outer membrane.

### c -di-GMP Analogs

#### 2′-F-c-di-GMP

Zhou et al. developed a potent DGC inhibitor by substituting 2′-OH of c-di-GMP to fluoride (Zhou et al., 2013). The chemical 2′-F-c-di-GMP (Figure 2B) binds to the I-site, and IC_50_ was 11 μM when it was tested with WspR, a DGC of *P. aeruginosa*. Interestingly, this chemical also inhibits PDEs, and the inhibitory activity against PDEs is stronger than that against the DGC, which could increase the cellular concentration of c-di-GMP. Unfortunately, this chemical is also not membrane-permeable like most other c-di-GMP and GTP analogs, so cell membrane-permeable derivatives are required to perturb cellular c-di-GMP signaling *in vivo*.

#### c-di-Inosinylic Acid

c-di-inosinylic acid (Figure 2C) was identified as an effective inhibitor against a DGC, Slr1143 of *Synechocystis* sp. after testing five structural analogs of c-di-GMP (Ching et al., 2010). c-di-inosinylic acid has a hypoxanthine base instead of the guanine base. For the analysis, each inhibitor was mixed with purified Slr1143, and its inhibitory effect on the c-di-GMP production by Slr1143 was determined by a high-performance liquid chromatograph (HPLC) assay. Since c-di-inosinylic acid is structurally similar to c-di-GMP, this analog is predicted to bind to the I-site of the DGC, Slr1143. c-di-Inosinylic acid has a stronger inhibitory activity than c-di-GMP with ~100 μM of IC_50_. This study, however, did not determine whether or not c-di-inosinylic acid inhibited the biofilm formation of *Synechocystis* sp.

#### Triazole-Linked Analogs

A target in the c-di-GMP structure for developing DGC inhibitors is the phosphodiester moiety. One of the benefits of the modification of the phosphodiester moiety is that it can confer the resistance to the hydrolysis by PDEs. Ferniclola et al. designed new molecules in which the phosphodiester moiety was replaced with isosteric non-hydrolyzable 1,2,3-triazole moiety. At the same time, the length between two guanine bases was varied by adding different triazole connectors (Fernicola et al., 2015). These molecules were synthesized as linear forms instead of cyclic forms because linear forms are easier to be synthesized chemically than cyclic forms. These synthesized molecules were tested for their ability to inhibit both PleD, a DGC of *C. crescentus* and RocR, a PDE of *P. aeruginosa*. From an *in vitro* analysis using purified enzymes, two molecules inhibiting the DGC were identified. However, among these two, only DCI058 did not significantly inhibit the PDE, RocR. DC1058 (Figure 2D) binds to the I-site of the DGC, and its inhibitory activity against DGC is comparable to 2′-F-c-di-GMP. The PleD activity with 100 μM of DC1058 and 100 μM of GTP is ~32% of that without DC1058. Even though DC1058 has promising characteristics as a drug for the inhibition of biofilm formation through the inhibitory effect against bacterial DGCs, it did not inhibit bacterial biofilm formation probably due to low membrane permeability. Thus, it needs to be chemically modified to improve membrane permeability.

To measure the inhibitory activity of the compounds toward DGCs or PDEs in this study, circular dichroism (CD) spectroscopy was used (Stelitano et al., 2013b). In the presence of manganese, c-di-GMP forms a dimer by intercalating each other. In this structure, four guanines are aligned, and this alignment increases dichroic signal that can be detected by CD spectroscopy. However, this dichroic signal increase does not occur along with the dimer formation of c-di-AMP, another common bacterial second signaling molecule.

### GTP Analogs

#### MANT-GTP, MANT-GTPγS, and TNP-GTP

Since 2′,3′-*O*-(2,4,6-trinitrophenyl) (TNP)- and 2′ (3′)-*O*-(*N*- methylanthraniloyl) (MANT)- substituted nucleotides are potent inhibitors of guanylyl and adenylyl cyclases, and the catalytic domain of DGCs is similar to the catalytic domain of mammalian adenylyl cyclases, Spangler et al. tested various MANT- and TNP- substituted nucleotides to identify if they were inhibitors against DGCs (Spangler et al., 2011). Among those nucleotide analogs, MANT-GTP (Figure 2E), MANT-GTPγS (Figure 2F), and TNP-GTP (Figure 2G) acted as potent inhibitors against an *E. coli* DGC, YdeH in *in vitro* enzyme activity assay, in which the enzymatic product c-di-GMP was quantified by HPLC-MS/MS (high-performance liquid chromatography-tandem mass spectroscopy) analysis. The IC_50_ values of these chemicals were below 1 μM. In a fluorometric analysis observing fluorescence resonance energy transfer (FRET) between tyrosine and tryptophan residues of YdeH and the MANT group, MANT-GTP closely interacted with YdeH. Even though the exact interaction mechanism has not been identified, the fluorometric analysis indicates that probably MANT-GTP interacts with the YdeH active site instead of GTP. If so, MANT-GTP not only interferes with c-di-GMP signaling, but it may also interfere with other pathways where GTP is involved *in vivo*. Membrane permeability of the MANT- or TNP- substituted GTP has not been tested, but they are probably not membrane-permeable when their chemical structures are considered.

### Small Synthetic Molecules

#### Ebselen

Ebselen (Figure 2H) was identified by a rapid and quantitative high-throughput screen based on the differential radial capillary action of ligand assay (DRaCALA) as an inhibitor of a DGC/c-di-GMP interaction (Lieberman et al., 2014). In this study, the NIH clinical collection 1 (NCC1) library was used to screen for inhibitors of c-di-GMP-binding to the I-site of DGCs, PelD, and WspR. The NCC1 library is comprised of molecules that have a history of use in human clinical trials (Austin et al., 2004). Ebselen reduces DGC activity by covalently modifying cysteine residues near the c-di-GMP binding pocket, and consequently inhibits c-di-GMP regulation of flagella-mediated motility and biofilm formation in *P. aeruginosa*. Favorably, ebselen does not bind to PDE or PilZ proteins from *P. aeruginosa*. The IC_50_ values of ebselen for PelD and WspR are about 5.0 and 13.6 μM, respectively. Ebselen has favorable drug-like properties and has been used in phase III clinical trials (Ogawa et al., 1999)

DRaCALA is based on the differential mobility of protein-bound and -free radiolabeled ligands on a nitrocellulose membrane (Roelofs et al., 2011). In this assay, each purified protein is mixed with ^32^P radiolabeled c-di-GMP, and spotted on a nitrocellulose membrane. Then the distribution of the radioactivity is imaged directly by a phosphorimager (autoradiography). If radiolabeled c-di-GMP binds to the protein, a clear spot appears because of the low mobility of the protein. Otherwise, c-di-AMP disperses, resulting in no clear spot but a gray background. One of the advantages of this assay compared to spectroscopic methods is that the interference caused by spectroscopic properties of molecules is eliminated by direct measurement of radioactivity.

#### Catechol-Containing Sulfonohydrazide Compounds

Fernicola et al. used a three-dimensional pharmacophore model to identify small-molecule inhibitors of the well-characterized DGC enzyme PleD of *C. crescentus* (Fernicola et al., 2016). In this study, the Ligandscout algorithm (Wolber and Langer, 2005) and the purchasable subset of the ZINC database (Irwin and Shoichet, 2005) were used for screening inhibitors. The crystal structure of PleD bound by the non-cleavable substrate analog GTP-α-S and a pharmacophore-based approach revealed that the key amino acids in PleD interacting with the substrate are F330, F331, D344, R366, and K442. Also, a magnesium ion interacts with the β-, γ-bisphosphate moiety in the binding pocket. The chemical structures from the ZINC data base compounds were examined to test whether or not the structures interact with the amino acids and the magnesium ion in the binding pocket of PleD using Molegro Visual Docker software (CLCbio). Seven molecules which were selected by this *in silico* analysis were tested *in vitro* for their ability to inhibit the activity of the purified DGC. In the assay using circular dichroism spectroscopy, two catechol-containing sulfonohydrazide compounds, Amb2250087 (Figure 2I) and Amb379455 (Figure 2J) were identified to inhibit PleD competitively. These compounds were predicted to interact with the amino acid residues, N335, D344, and R366 and the magnesium ion in the binding pocket. The IC_50_ values of these compounds are both ~11 μM. These two inhibitors are found to bind to the catalytic site, not the I-site of the DGC. Previously, c-di-GMP analogs had been synthesized and tested for their ability to bind to the I site and inhibit DGC enzymes, and these compounds are likely ineffective against those DGCs without an I-site. However, since the catechol-containing sulfonohydrazide compounds directly inhibit the catalytic site, it might be effective to most DGCs. Unfortunately, these compounds do not reduce the intracellular level of c-di-GMP in bacteria probably due to a membrane permeability issue, so further chemical modifications are necessary to improve the delivery of these compounds inside cells.

#### Sulfasalazine and Eprosartan

Recently, Wiggers et al. screened 1500 FDA-approved drugs in the DrugBank database through a virtual screening method to find competitive inhibitors binding to the A-site of the DGC, *C. crescentus* PleD (Wiggers et al., 2018). The authors used the combination of the molecular docking strategy and ligand-based methods. In the molecular docking strategy, among the nine amino acid residues involved in the recognition of the GTP substrate in the structure of DGCs, two highly conserved residues playing an important role in the specificity to GTP over other nucleotides, N^335^ and D^344^ were set as constraints. In the ligand-based methods which search for the compounds similar to GTP, the distribution of shape and electrostatics similarity of compounds were analyzed. Among the top 200 candidates from the analysis, 10 compounds were selected based on the properties of the compounds such as A-site occupancy, constraint matching, hydrogen bonding network, and molecular diversity.

After the screening, the authors tested the top 10 candidates with *in vitro* enzyme assay using purified DGCs, WspR from *P. aeruginosa* and YdeH from *E. coli*, and discovered four chemicals inhibiting the activity of the DGCs. Further binding study with a mass spectrometry analysis revealed that the inhibition occurred through interacting with the active site in the GGDEF domain. Among those four, two had favorable biological properties as DGC inhibitors such as no inhibition of bacterial growth and no signs of interference with other metabolic pathways. Those two inhibitors are the anti-inflammatory sulfasalazine (Figure 2K) and the anti-hypersensitive eprosartan (Figure 2L). They inhibit the DGC activity in the micromolar range. The IC_50_ values of sulfasalazine are 200 μM for YdeH and 360 μM for WspR, and those of eprosartan are 888 μM for YdeH and 170 μM for WspR, respectively. The big difference between the IC_50_ values of eprosartan for the different DGCs indicates that not only the key conserved residues interacting with the substrate GTP in DGCs but also different A-site peripheral residues should be considered for designing a virtual inhibitor screening to discover inhibitors with a broad DGC spectrum. Both compounds reduce aggregation of *E. coli* in the culture broth, suggesting that these two chemicals have anti-biofilm property *in vivo* (Wiggers et al., 2018).

#### N-(4-anilinophenyl)benzamide

A high throughput screen using a bacterial reporter assay identified seven DGC inhibitors (Sambanthamoorthy et al., 2012). The *V. cholerae* reporter system used in the study is consisted of two plasmids, one of which contains luciferase reporter genes (*lux* genes) and the other a DGC enzyme gene, respectively. A luciferase gene is transcriptionally fused with the promoter of the *VC1673* gene of *V. cholerae* whose transcription is induced by c-di-GMP. The second plasmid encodes a DGC enzyme gene to increase the intracellular c-di-GMP level. Out of 66,000 compounds/natural product extracts at the Center for Chemical Genomics at the University of Michigan, the authors identified 7 small molecule compounds that inhibited at least two DGC enzymes, VC2370 from *V. cholerae* and WspR from *P. aeruginosa* after determining IC_50_, toxicity to eukaryotic cells, inhibition of bacterial growth, etc. In addition, some inhibitors inhibited the formation of *V. cholerae* biofilm and/or *P. aeruginosa* biofilm. Especially, N-(4-anilinophenyl)benzamide (Figure 2M) is effective against both biofilms and decreases *in vivo* c-di-GMP concentration, which makes this compound one of the most promising antibiofilm drugs among the seven inhibitors. However, it does not disperse a preformed biofilm. The IC_50_ values of the compound is 1 μM for VC2370 and 17.83 μM for WspR.

#### [2- oxo-2-(2-oxopyrrolidin-1-yl)ethyl] 1,3-benzothiazole-6-carboxylate and 4-(2,5-dimethylphenoxy)-N-(4-morpholin-4-ylphenyl)butanamide

Recently, a FRET (Foster resonance energy transfer)-based assay that can measure a cellular c-di-GMP concentration was developed (Christen et al., 2010). For the construction of this FRET-based activity assay system, mYPet and cCYPet fluorescence proteins were attached at the N- and C-terminus of the c-di-GMP binding domain of the PilZ protein, YcgR. When the YcgR domain binds c-di-GMP, the angle of the two fluorescent proteins each other changes due to the conformational change of the YcgR domain, then the fluorescence property reflecting c-di-GMP levels (535/470 nm) changes. Using this assay, Christen et al. screened a compound library containing 27,502 commercially available small chemicals to discover inhibitors of a DGC, *C. crescentus* DgcA (Christen et al., 2019). The authors first identified 49 compounds as potent inhibitors. After secondary assay considering IC_50_, cytotoxicity, and easiness of chemical synthesis and modification, two compounds were selected as promising DGC inhibitors, which were [2- oxo-2-(2-oxopyrrolidin-1-yl)ethyl] 1,3-benzothiazole-6-carboxylate (Figure 2N) and 4-(2,5-dimethylphenoxy)-N-(4-morpholin-4-ylphenyl)butanamide (Figure 2O). Their IC_50_ values are 4.0 and 6.4 μM for the *C. crescentus* DGC enzyme DgcA, respectively. When the scaffolds of the two compounds were chemically modified to produce 44 derivatives, more than half of them acted as allosteric inhibitors.

#### N′-((1E)-{4-ethoxy-3-[(8-oxo-1,5,6,8-tetrahydro-2H-1,5-methanopyrido[1,2-a] [1,5]diazocin-3(4H)-yl)methyl]phenyl}Methylene)-3,4,5-Trihydroxybenzohydrazide

Another *in silico* virtual screening performed by the group discovered N-(4-anilinophenyl)benzamide led the discovery of several potent DGC inhibitors (Sambanthamoorthy et al., 2014). For this virtual screening, two pharmacophore data were used: one generated based on the interaction between the amino acid residues of the *C. cescentus* DGC PleD and a guanine base and the other based on the oroidin template containing some of the features of the guanine base. By using these two pharmacophores, a chemical library from a database of commercially available compounds was generated, and 250 compounds in the library were experimentally analyzed. For DGC inhibition assay, the conversion of GTP to c-di-GMP by DGCs, *Thermotoga maritima* tDGC and *P. auruginosa* WspR, was monitored by measuring the amount of pyrophosphate produced during the c-di-GMP synthesis reaction. Out of 250 compounds they tested, 4 compounds showed significant inhibitory activity. Among those 4 compounds, LP3134, N′-((1E)-{4-ethoxy-3-[(8-oxo-1,5,6,8-tetrahydro-2H-1,5-methanopyrido[1,2-a] [1,5]diazocin-3(4H)-yl)methyl]phenyl}methylene)-3,4,5-trihydroxybenzohydrazide (Figure 2P) was the most promising biofilm inhibitor because it inhibited the biofilm development of both *P. aeruginosa* and *Acetobacter baumannii* under both static and flow conditions. In addition, it showed the lowest cytotoxicity to human keratinocytes; it did not show any cytotoxicity below 300 μM, which is much higher than the IC_50_ value for WspR, 45 μM.

## Discussion

Diverse approaches have been used to discover DGC inhibitors so far. Those approaches are: (1) Chemical modification of the substrate GTP. This method screens competitive inhibitors of DGCs that bind to the active site A-site. Since the inhibitors can be structurally similar to the substrate GTP, a caveat of this approach is that they might interfere with other GTP-involved metabolic pathways in bacteria or host cells. Thus, this aspect should be tested when they are considered as antibiofilm drugs. (2) Chemical modification of the DGC product, c-di-GMP. Many DGCs have the I-site that provides a feedback inhibition by binding the enzyme's product c-di-GMP. Most c-di-GMP analog inhibitors bind the allosteric inhibitory site I-site, and act as non-competitive inhibitors. Generally, the inhibitors binding to the I-site are not effective to DGCs with no I-site. (3) Chemical modification of backbone molecules similar to the structure of c-di-GMP. c-di-GMP analogs can be degraded by PDEs. To avoid this problem, scientists designed non-cyclic (for easy chemical synthesis) backbone molecules similar to c-di-GMP, and tested their chemical derivatives for inhibitory activity against DGCs. This type of chemical generally acts as non-competitive inhibitors by binding to the I-site. (4) High throughput screening of chemical libraries through *in vitro* assays. Chemical libraries provided for research by NIH, FDA, university centers, etc. have been tested for inhibitory activity against DGCs. For *in vitro* high throughput screening, scientists developed *in vitro* assay methods that can measure the binding of c-di-GMP to purified DGCs by FRET (Foster Resonance Energy Transfer) (Christen et al., 2019) or DRaCALA (Differential Radial Capillary Action of Ligand Assay) (Lieberman et al., 2014). (5) High throughput screening of chemical libraries using *in vivo* assays. An ideal high throughput screening for chemical inhibitors of DGCs would incorporate a system that monitors the changes of cellular c-di-GMP concentration *in situ*. This kind of assay methods determines its efficacy on whole bacteria, including membrane permeability. One example is to develop a luciferase reporter system responding to a cellular c-di-AMP concentration *in situ* (Sambanthamoorthy et al., 2012). However, this method does not always find inhibitors of DGCs. When a chemical lowers cellular c-di-AMP concentration, this can be caused by an inhibitory effect of the chemical on DGC activity directly or inhibitory effect on other metabolic pathways influencing cellular c-di-AMP concentrations, including pathways affecting GTP synthesis. This aspect should be tested after screening. (6) High throughput screening of chemical libraries through *in silico* methods followed by *in vitro* assays. This method uses computer algorisms to screen small molecule databases. For this method, the structure of DGCs bound by GTP (targeting the A site) or c-di-GMP (targeting the I-site) must be determined by X-ray crystallography or NMR. Based on the binding patterns of the ligands to DGCs, it is determined if a chemical has the potential to bind to DGCs. Since there are several computer algorithms developed already, this method can be an approach for initial screening of various chemicals if crystal structures of ligand-bound target proteins are available. Some computer algorisms for this type of screening are collected in websites, such as click2drug.org.

The genomes of most gram-negative bacteria encode multiples of GGDEF domain-containing proteins. For example, total 34 GGDEF domain proteins (18 GGDEF domain proteins and 16 GGDEF-EAL domain proteins) are encoded on the chromosome of *P. aeruginosa* (Valentini and Filloux, 2016) and 12 GGDEF domain proteins on the chromosome of *Salmonella enterica serovar* Typhimurium (Romling et al., 2005). It was shown that multiple DGCs affect biofilm formation, so an inhibitor inhibiting only one DGC might not affect biofilm formation (Kulasakara et al., 2006). The inhibitory activity of the discovered DGC inhibitors has been tested using only one or two DGCs. Thus, further studies are necessary to figure out whether or not these inhibitors show a broad-spectrum inhibitory activity toward diverse DGCs before being developed as antibiofilm drugs.

Each microorganism has a unique cell wall structure, which might confer different penetrability to each chemical. Currently, however, there are very little data on what microorganisms are affected by the discovered DGC inhibitors in terms of biofilm formation, which should be tested in the future.

Although several types of DGC inhibitors are identified, precise molecular actions of most inhibitors are not well-determined. Further studies such as determining the structures of inhibitor-bound DGCs will facilitate to design derivatives with better inhibitory ability.

## Conclusion

c-di-GMP plays an essential role in biofilm formation in diverse bacteria, so targeting c-di-GMP signaling appears to be a promising approach to control bacterial biofilm formation. The discovery of novel DGC inhibitors has been greatly assisted by the development of assays suitable for high-throughput screening (HTS) of chemical compounds. HTS should be based on reliable assays and, whenever possible, performed using living cells along with purified enzymes to select membrane-permeable compounds. The high throughput methods used to identify DGC inhibitors can also be used to discover di-adenylate cyclase (DAC) inhibitors. Many gram-positive bacteria including firmicutes and some gram-negative bacteria produce cyclic di-adenylate monophosphate (c-di-AMP) instead of or accompanying with c-di-GMP. c-di-AMP is also essential for biofilm formation by some pathogenic bacteria (Corrigan et al., 2011; Du et al., 2014; Cheng et al., 2016; Peng et al., 2016; Fahmi et al., 2019). It is expected that more potent DCG inhibitors with better druggable chemicals will be identified or designed in the near future.

## Author Contributions

KC designed and wrote the manuscript, made the figures and added information to the table. RT wrote the manuscript and made the table. J-HK added information to the manuscript.

## Conflict of Interest

The authors declare that the research was conducted in the absence of any commercial or financial relationships that could be construed as a potential conflict of interest.
